# A quasi-reagentless point-of-care test for nitrite and unaffected by oxygen and cyanide

**DOI:** 10.1038/s41598-019-39209-y

**Published:** 2019-02-22

**Authors:** Tiago Monteiro, Sara Gomes, Elena Jubete, Larraitz Añorga, Célia M. Silveira, Maria Gabriela Almeida

**Affiliations:** 1 UCIBIO, REQUIMTE, Faculdade de Ciências e Tecnologia, Universidade NOVA de Lisboa, 2829-516 Monte de Caparica, Portugal; 2 CIDETEC, Sensors Unit, Parque Científico y Tecnológico de San Sebastián, P° Miramón 196, 2014 Donostia, San Sebastián, Spain; 3 Instituto de Tecnologia Química e Biológica António Xavier, Universidade NOVA de Lisboa, Av. da República, 2780-157 Oeiras, Portugal; 4 Centro de Investigação Interdisciplinar Egas Moniz (CiiEM), Instituto Superior de Ciências da Saúde Egas Moniz, Campus Universitário, Quinta da Granja, 2829-511 Caparica, Portugal

## Abstract

The ubiquitous nitrite is a major analyte in the management of human health and environmental risks. The current analytical methods are complex techniques that do not fulfil the need for simple, robust and low-cost tools for on-site monitoring. Electrochemical reductase-based biosensors are presented as a powerful alternative, due to their good analytical performance and miniaturization potential. However, their real-world application is limited by the need of anoxic working conditions, and the standard oxygen removal strategies are incompatible with point-of-care measurements. Instead, a bienzymatic oxygen scavenger system comprising glucose oxidase and catalase can be used to promote anoxic conditions in aired environments. Herein, carbon screen-printed electrodes were modified with cytochrome *c* nitrite reductase together with glucose oxidase and catalase, so that nitrite cathodic detection could be performed by cyclic voltammetry under ambient air. The resulting biosensor displayed good linear response to the analyte (2–200 µM, sensitivity of 326 ± 5 mA M^−1^ cm^−2^ at −0.8 V; 0.8–150 µM, sensitivity of 511 ± 11 mA M^−1^ cm^−2^ at −0.5 V), while being free from oxygen interference and stable up to 1 month. Furthermore, the biosensor’s catalytic response was unaffected by the presence of cyanide, a well-known inhibitor of heme-enzymes.

## Introduction

Nitrite (NO_2_^−^) is an inorganic anion that is found ubiquitously in food, drinking water and the environment, originating from either the biogeochemical nitrogen cycle or from anthropogenic input. Excessive exposure to this anion may present serious risks to public health^1,2^ and ecological systems^3^. Therefore, the analytical surveillance of NO_2_^−^ is crucial in the management of health and environmental risks. From a clinical diagnosis perspective, NO_2_^−^ is an important indicator of urinary tract infection (cystitis) due to high levels being present in the urine upon conversion of nitrate by bacterial nitrate reductases^4^. It is also a marker for constitutive oxygen-dependent nitric oxide (NO) synthase activity and endothelial function in humans, with decreased plasma NO_2_^−^ levels being correlated with increasing numbers of cardiovascular risk factors^5,6^. Furthermore, it has been proposed that NO_2_^−^ is a constitute intravascular storage and delivery source of NO, a potent cardioprotective-signalling molecule. This association is of great interest in biomedical research, since administration of NO_2_^−^ could potentially have therapeutic effects in situations where the oxygen-dependent enzymatic production of NO is compromised (i.e. ischemia)^5,7,8^.

**Figure 1:**
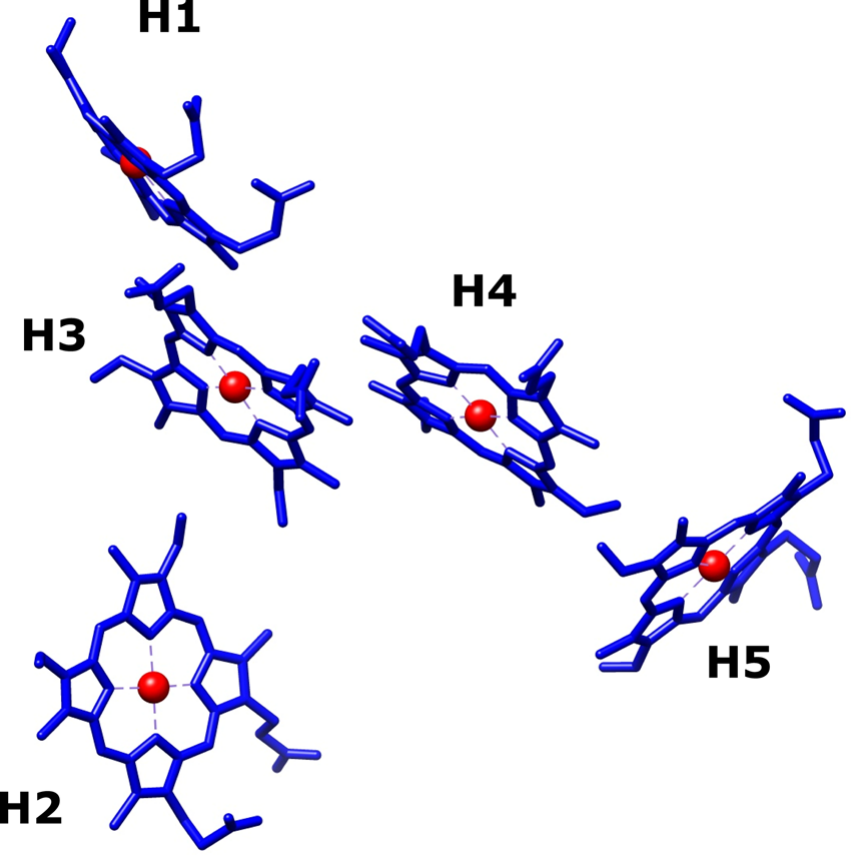
Heme groups of the NrfA subunit from *D. desulfuricans* ATCC 2774. Midpoint reduction potentials^17^ (*vs* SHE) of the individuals hemes (H) are as follow: H1 −80 mV, H2 −50 mV, H3 −480 mV, H4 −400 mV, H5 + 150 mV. The figure was prepared with the software UCSF Chimera version 1.13.1 using the RCSB PDB entry 1OAH.

Most of the existing methods for NO_2_^−^ monitoring^9^ are labour-intensive, require expensive laboratory equipment’s and/or skilled personnel, and therefore, they cannot fulfil the demand of simple, fast, accurate, low-cost and on-field or point-of-care (POC) detection that the environmental, food and clinical industries need. In this context, electrochemical biosensors based on reductase enzymes are presented as a powerful alternative to the existing methods, due to their fast response time, high selectivity and sensitivity, and miniaturization potential^10^. The multihemic cytochrome *c* nitrite reductase (c*c*NiR) from *Desulfovibrio desulfuricans* ATCC 27774 has been used as the key biorecognition element in the development of such devices^11–15^. This enzyme performs the six-electron reduction of NO_2_^−^ to ammonia and is comprised of a pentahemic catalytic subunit NrfA (61 kDa) bound to a tetrahemic electron donor subunit NrfH (19 kDa), in the proportion of 2NrfA:1NrfH. All hemes are *c*-type hexa-coordinated, except for the active centre *c*-heme, which is penta-coordinated with the sixth axial position vacant^16,17^. The hemes in the catalytic subunit exhibit a broad range of reduction potentials, that span from −0.48 to + 0.15 V *vs* SHE (Fig. 1)^17^.

Despite their advantages, the real-world application of biosensors based on reductase enzymes is limited by the need of anoxic working conditions. Molecular oxygen (O_2_) is a main interferent in the analytical process because its reduction to hydrogen peroxide (H_2_O_2_) generates an intense cathodic current that can mask important redox processes that occur at very low potentials (−0.2 and −0.8 V *vs* SHE). This is the case of the catalytic reduction of NO_2_^−^ by c*c*NiR^13–15^. Moreover, O_2_ can react with redox mediators in their reduced form^18,19^ and, in the case of other oxidoreductase-based biosensors, O_2_ can even compete with them for the enzyme redox co-factors^19^.

The standard strategies for O_2_ removal employed in laboratory settings, such as argon purging or vacuum degassing, are incompatible with on-site monitoring and point-of-care testing. Furthermore, they are not feasible when handling a large number of test samples and can lead to the formation of foam in biological samples. Alternatively, chemical O_2_ scavengers, such as sodium sulfite^20^, can be used to achieve the desired deoxygenation without being cumbersome or compromising the sample’s integrity. Nonetheless, it has been reported that c*c*NiR catalyses the reduction of sulfite to sulfide^21^, rendering this chemical species incompatible with NO_2_^−^ reductase-based electrochemical biosensing. However, a well-known bienzymatic O_2_ scavenging system based on the combined action of glucose oxidase (GOx) and catalase (Cat) can be employed to efficiently deoxygenate a sample^22–24^. Both enzymes are free in solution, and upon addition of glucose (the main substrate), O_2_ is consumed in a two-step cycle. As long as the main substrate is present, and GOx/Cat remain active, any atmospheric O_2_ that diffuses into the aqueous phase is rapidly scavenged. In this manner, anoxic conditions can be maintained for extended periods of time in an open-air environment in small sample volumes (100–200 µL)^23^.

Following this bienzymatic strategy for sample deoxygenation, we have previously developed a miniaturized electrochemical biosensor based on c*c*NiR for the detection of NO_2_^−^ in real samples^15^. The biosensor was capable of operating in anoxic conditions for 1 hour, in an open-air environment. Still, despite the good analytical performance of the device, the GOx and Cat enzymes were employed free in solution. This is unsuitable for a completely reagentless biosensing device and therefore, for its future commercialization. To face this problem, in this paper, we present an improved prototype fabricated by using a very simple co-immobilization procedure of c*c*NiR, GOx and Cat on unmodified carbon screen-printed electrodes (SPEs). The analytical performance and the long-term stability of the new biosensor were evaluated by cyclic voltammetry (CV). Additionally, the catalytic activity of the new biosensor in the presence of cyanide (CN^−^), a well-known inhibitor of heme-enzymes, like Cat and c*c*NiR^25,26^, that can be present in biological fluids due to dietary habits and smoking activities, among other sources^27,28^, was also analysed.

By immobilizing the scavenger system together with the c*c*NiR enzyme on SPEs, this work represents a step forward in the establishment of a truly disposable methodology for on-site NO_2_^−^ monitoring. Furthermore, the immobilized scavenger system could also be coupled with other reductases, opening a whole new world of possible POC tests.

## Materials and Methods

### Reagents and solutions

Glucose, hydrochloric acid and Trizma^®^ were purchased from Sigma-Aldrich. Potassium chloride and sodium nitrite were obtained from VWR. Potassium cyanide was purchased from Merck. All reagents were of analytical grade. Solutions were prepared with deionized water (18 MΩ cm) from a Millipore MilliQ purification system.

GOx (Type II from *Aspergillus niger* 19.3 U mg^−1^) and Cat (from bovine liver, 2–5 kU mg^−1^) were purchased as lyophilized powders from Sigma and solutions were prepared in 100 mM Tris-HCl pH 7.6 buffer, with 100 mM KCl.

c*c*NiR (300 U mg^−1^) was purified from *D. desulfuricans* ATCC 27774 cells, as reported by Almeida and co-workers^17^. The protein concentration was 0.8 mg mL^−1^ in 50 mM phosphate pH 7.6 buffer.

### Biosensors preparation

The disposable SPEs consisting of a carbon working electrode (WE, *ϕ* = 4.4 mm), a carbon counter electrode and a silver/silver chloride (Ag/AgCl) pseudoreference electrode (0.302 V *vs* SHE) were produced at CIDETEC using a Thieme 110E screen-printing machine from Thieme GmbH&Co (Teningen, Germany), an UV tabletop dryer Aktiprint T/A 40-2 from Technigraf (Hessen, Germany) and an oven PN 200 from Carbolite (Derbyshire, UK)^29,30^.

The SPEs were used as provided without pre-activation. The WEs were coated with a 5 µL drop of a c*c*NiR solution and air dried for 40 minutes at room temperature (22 ± 2 °C). Afterwards, a 4 µL drop of GOx (187.5 U mL^−1^) and Cat (25 kU mL^−1^) mixture was placed on the c*c*NiR-coated WE and the modified-electrode was air dried for another 40 minutes. The resulting biosensors were stored dry at 4 °C until use.

In the assays where home-made pyrolytic graphite electrodes (PGE) were used instead of SPE, the carbon surface was properly cleaned prior to any modification. The WEs (*ϕ* = 3 mm) were polished with alumina 0.3 µm for 2 minutes and then thoroughly rinsed with ethanol 96% (v/v) and deionized water. The electrodes were then sonicated in deionized water for about 5 minutes, being thoroughly rinsed afterwards and dried with an air stream. Once cleaned, the PGEs were covered with a 5 µL drop of a c*c*NiR solution and left to dry for 40 minutes at room temperature. Before placing the WEs in the electrochemical cell, they were rinsed with 100 mM Tris–HCl pH 7.6 buffer containing 100 mM KCl.

### Electrochemical measurements

All the electrochemical experiments were performed with a PSTAT 12 potentiostat from Autolab (KM Utrecht, The Netherlands), using the software GPES 4.9. A DropSens DSC boxed connector was used to connect the SPE to the potentiostat.

The electrochemical measurements of the SPE-based biosensors were performed by covering the three-electrode system with 50 µL of solutions prepared in the supporting electrolyte (100 mM Tris–HCl pH 7.6 buffer with 100 mM KCl and 80 mM glucose).

For the PGE-based biosensors, the electrochemical measurements were carried out using an Ag/AgCl electrode and Pt wire (both from Radiometer) as reference and counter electrodes, respectively. In addition, the enzymes GOx and Cat were added to 5 mL of the supporting electrolyte in the following final concentrations: 15 U mL^−1^ and 2 kU mL^−1^, respectively.

The CVs were plotted at room temperature (22 ± 2 °C), with a scan rate of 20 mV s^−1^, from −0.1 V to −0.8 V (*vs* reference system). All current values were determined using the analysis software QSoas 1.0^31^.

### Cyanide interference

The performance of the SPE and PGE-based biosensors in the presence of CN^−^ was assessed by spiking the supporting electrolyte with 10 µM of NO_2_^−^ and afterwards with 10 µM of the interfering compound. A 5 min incubation period (without stirring) was observed between additions. All solutions were prepared in 100 mM Tris-HCl pH 7.6 buffer, with 100 mM KCl.

The catalytic currents (ΔI_cat_) were determined at the cathodic peak (ca. −0.4 V); all values were corrected for the non-catalytic current measured in the absence of NO_2_^−^. The relative catalytic response (Equation 1) was calculated as:

1 $$Relative\,Catalytic\,Response\,({\rm{ \% }})=\frac{{\rm{\Delta }}{I}_{catN{{O}_{2}}^{-}{\rm{\& }}C{N}^{-}}}{{\rm{\Delta }}{I}_{catN{{O}_{2}}^{-}}}\times 100$$

Controls were performed by spiking the supporting electrolyte (containing NO_2_^−^) with equal volumes of buffer solution. All assays were replicated three times (*n* = 3).

### Analytical performance – sensitivity, stability and reproducibility

The sensitivity (slope of the calibration curve) of the SPE-based biosensor was determined by measuring the response to different NO_2_^−^ standard solutions (one electrode per standard), with concentrations ranging from 0.8 to 200 µM. A 5 min incubation period was observed before recording the CV. The ΔI_cat_ values were determined at −0.5 V and −0.8 V and plotted *vs* the analyte concentrations. Each assay was replicated three times (*n* = 3).

The SPE-based biosensor’s long-term stability was evaluated for 1 month. A batch of biosensors was prepared as previously described and stored dry at 4 °C when not in use. On the first day, 6 single-use biosensors were used to measure the initial response to a 50 µM NO_2_^−^ standard solution. Afterwards, the measurements were repeated 3 times every five days, up to 30 days.

The reproducibility was determined by calculating the relative standard deviation (RSD) of the ΔI_cat_ of 10 SPE-based biosensors to 50 µM NO_2_^−^.

### Real sample analysis

Urine was collected from a healthy male volunteer (with informed consent) 4 hours after the first morning evacuation using a clean plastic vial and no pre-treatment was performed. The pH was measured using a 210 Benchtop pH/mV meter (Bante Instruments). Immediately prior to the analysis, 820 µL of urine were supplemented with 80 µL of glucose (1 M) and 100 µL of NO_2_^−^ standard (0–2 mM) prepared in distilled water. Afterwards, 50 µL of the mix were placed on the SPE-based biosensors and a 5 min incubation period was observed before recording the CV. Each assay was replicated three times (*n* = 3).

## Results and Discussion

### Immobilization of the oxygen scavenger system

**Figure 2:**
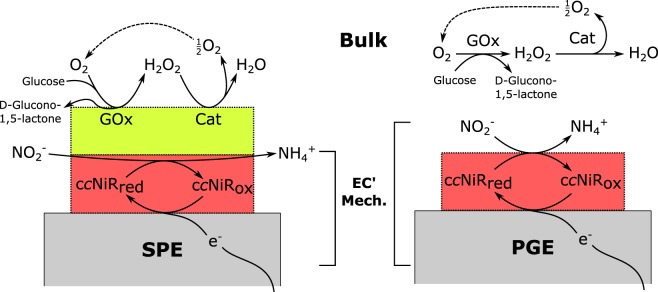
Schematic representation of the c*c*NiR/GOx/Cat-modified SPE and c*c*NiR-modified PGE, with GOx and Cat in solution. *EC’ Mech*. stands for the catalytic reaction mechanism where c*c*NiR is first reduced (c*c*NiR_red_) by the WE in the electrochemical reaction (E), and afterwards it is reoxidized (c*c*NiR_ox_) in the chemical reaction (C’) with NO_2_^−^.

In our previous work^15^, the GOx/Cat O_2_ scavenger system was successfully employed to remove dissolved O_2_ from the supporting electrolyte, maintaining anoxic conditions in the electrochemical cells. Since c*c*NiR was immobilized on the surface of carbon SPE, the analyte detection was easily achieved by spiking the supporting electrolyte with NO_2_^−^ solutions and recording the corresponding ΔI_cat_ increase by CV. However, the reported system required the addition of the GOx and Cat enzymes to a relatively high working volume (5 mL) of supporting electrolyte, increasing the overall cost of the assay. To face these problems, a new prototype has been developed in which the GOx and Cat are immobilized together with the c*c*NiR on the SPE and the volume of the supporting electrolyte is reduced down to 50 µL. To ensure direct electron transfer between the WE and c*c*NiR, the enzyme needs to be in contact with the surface of the electrode^10,32^. For this reason, the sensing layer (where NO_2_^−^ reduction occurs) was the first to be adsorbed on the electrode surface, followed by the immobilization of the GOx/Cat layer, as schematized in Fig. 2 for the SPE.

**Figure 3:**
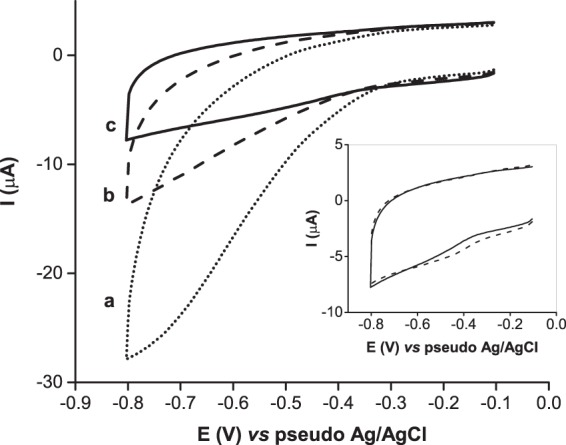
CVs of the GOx and Cat-modified carbon SPEs: (**a**) background current recorded in the supporting electrolyte without glucose (O_2_ is present); (**b**) background current recorded after spiking the supporting electrolyte with 80 mM glucose (t_0_ min); (**c**) background current recorded in the supporting electrolyte containing 80 mM glucose after 5 min. Inset: background current after (line) 5 and (dash) 60 minutes.

To analyse the ability of the GOx and Cat enzymes to promote anoxic conditions in an aerated environment once immobilized, the electrodes were modified with both enzymes but without c*c*NiR. The resulting GOx/Cat-modified SPEs were covered with 50 µL of the supporting electrolyte and characterized by CV. As shown in Fig. 3, in the absence of glucose, a broad cathodic wave is observed due to the electrochemical reduction of dissolved O_2_ in the supporting electrolyte. After adding glucose to the electrolyte, the cathodic current attributed to O_2_ reduction drastically decreases. This is due to glucose being oxidized by GOx, while the co-substrate O_2_ (electron acceptor) is reduced to H_2_O_2_. The latter is then dismutated by Cat into O_2_ and water (Fig. 2). Despite O_2_ regeneration at the end of the cycle, for each iteration, the total amount of the co-substrate is reduced by a factor of 2^23^. Owing to the high turnover numbers of GOx and Cat, after a few cycles, the O_2_ content quickly drops below the detection limit, and the background current remains stable for one hour (cf. Fig. 3, inset). The results obtained are comparable to those reported in previous works^15,23^, where GOx and Cat were used free in the supporting electrolyte. Hence, the immobilization of both enzymes on a carbon SPE does not compromise their bioactivity.

### Cyanide interference

**Figure 4:**
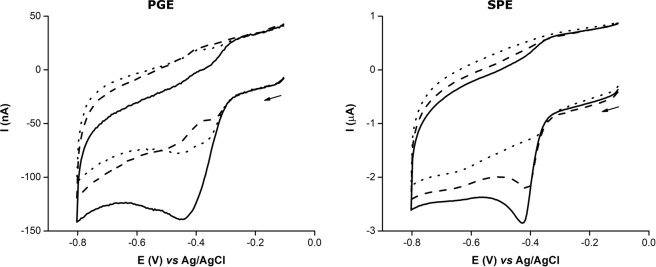
CVs obtained for the c*c*NiR-modified PGE (GOx and Cat in solution) and c*c*NiR/GOx/Cat-modified SPE in the presence of 10 µM NO_2_^−^ before (—) and after (╸╸╸) adding 10 µM CN^−^ to the solution. The non-catalytic (⋅⋅⋅) current (without NO_2_^−^/CN^−^ in solution) for each electrode is also shown. Supporting electrolyte 100 mM Tris-HCl (pH 7.6) with 100 mM KCl and 80 mM glucose; scan rate 20 mV s^−1^.

CN^−^ is a well-known inhibitor of heme-enzymes^25,26^ and its effect on the biosensor’s catalytic response was measured in the presence of equimolar concentration of NO_2_^−^. Figure 4 shows the CVs obtained for the assays with c*c*NiR/GOx/Cat-modified SPE and, for comparison, the ones obtained for the c*c*NiR-modified PGE (in which the GOx and Cat were added to the supporting electrolyte). In both cases, upon the addition of NO_2_^−^, a well-defined cathodic peak was obtained around −0.4 V (NO_2_^−^ bioelectroreduction). This was due to the direct electron transfer between c*c*NiR and the WE surface, coupled with the enzyme catalysed 6 electron reduction reaction of NO_2_^−^ to ammonia, according to a catalytic (EC’) mechanism, in which a reversible electron transfer reaction is followed by an irreversible chemical reaction (Fig. 2)^10,33,34^.

**Figure 5:**
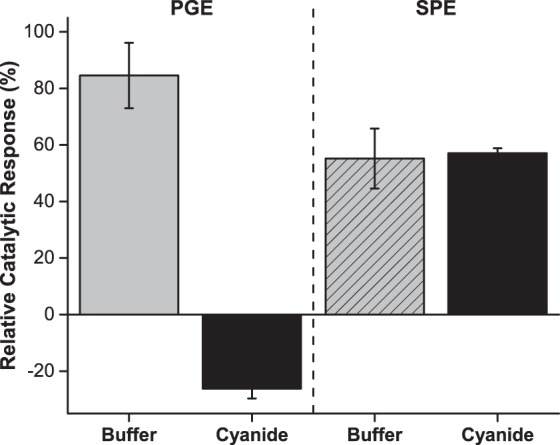
Relative catalytic response of the PGE and SPE-based biosensors to CN^−^ interference (*n* = 3). NO_2_^−^ was previously added to the supporting electrolyte; the analyte-interferent ratio was 1:1. Control was performed by spiking the supporting electrolyte with a CN^−^ free buffer solution.

In the presence of CN^−^, the cathodic peak current obtained for the c*c*NiR-modified PGE showed a drastic decrease bellow the non-catalytic current, accompanied by a + 90 mV shift in the peak potential. A similar shift (+70 mV) was observed in non-turnover conditions for the c*c*NiR from *Shewanella oneidensis*, which was attributed to the binding of the ligand to the penta-coordinated catalytic heme^35^. It was also reported that the binding event of CN^−^ to the c*c*NiR from *Escherichia coli* resulted in a decrease in catalytic current and a displacement of the peak potential towards more positive values^26^. A more drastic reduction potential shift was observed in the presence of carbon monoxide (another inhibitor of c*c*NiR), where the binding event resulted in the dislodgment of the catalytic heme reduction potential to outside of the voltammogram envelop^36^. Therefore, the binding of CN^−^ to several of the heme groups from the c*c*NiR complex (see Fig. 1), particularly to the catalytic heme, could be shifting its reduction potential outside the range of applied potentials, explaining why the cathodic peak around −0.4 V was smaller than the one observed in the non-catalytic voltammogram. However, for the modified SPE, the decrease of the cathodic peak current and shift in peak potential (+10 mV) were far smaller. Considering the addition of equal volumes of CN^−^ or buffer (control) solutions caused a similar decrease in current response (about 50%; see Fig. 5), we conclude that this was due to the dilution of NO_2_^−^ and, therefore no inhibitory effects of CN^−^ were observed on the SPE based biosensor. Note that for the modified PGE, the catalytic response decreased 15% due to NO_2_^−^ dilution, but the enzyme was completely inhibited upon addition of CN^−^, since the relative catalytic response decreased 120%. This value resulted from the current response in the presence of the inhibitor being smaller than the initial non-catalytic current, as observed in Fig. 4.

Why c*c*NiR was not effectively inhibited when it was immobilized on the SPE is not clear, but could be due to several reasons: (i) CN^−^ adsorption by the carbon paste of the SPE; (ii) CN^−^ binding to the Cat enzyme; (iii) the formation of glucose-cyanohydrin. Firstly, the carbon ink used in the fabrication of the SPE could produce a unique environment that prevented the inhibitor from successfully binding to the NO_2_^−^ reductase active site. Activated carbon has been shown to adsorb CN^− ^^37^; the ink used in the fabrication of the printed working and counter electrodes might behave as activated carbon, adsorbing CN^−^ to some extent. Secondly, Cat could be scavenging CN^−^, since the latter binds to the active site of the enzyme^25^. Thus, the inhibitor that reached the c*c*NiR layer on the SPE could be insufficient to effectively block its activity. However, enzyme inhibition was observed in the PGE assay, where the Cat/CN^−^ ratio was kept the same; so the hypothesis of Cat acting as a CN^−^ scavenger seems unlikely. Finally, the glucose present in the support electrolyte could be acting as a CN^−^ scavenger, producing glucose-cyanohydrin^38^. However, this possibility was ruled out, as this was not observed in the assays with the PGE. Therefore, the c*c*NiR enzyme was shielded from inhibition probably due to the adsorption of CN^−^ by the carbon surfaces of the SPE.

Worth of mention, no O_2_ or H_2_O_2_ interferences were observed on the CVs after spiking the electrolyte with CN^−^, which means that the immobilized GOx and Cat remained active in the presence of CN^−^. Since no inhibition in reductase and catalase activities were observed when using the SPE as immobilization surface, it is reasonable to say that this system could be used with other heme-proteins based biosensing applications, such as other cytochromes and hemoglobins.

### Nitrite detection

**Figure 6:**
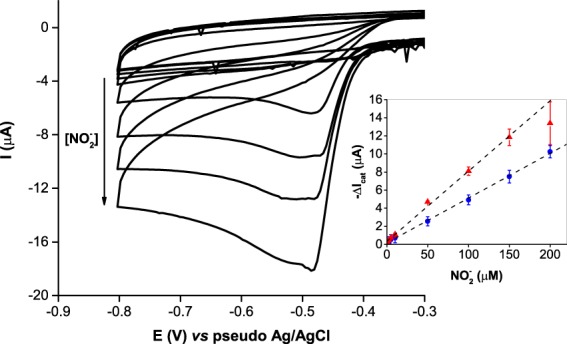
CVs of the SPE-based biosensors response to NO_2_^−^ standard solutions (0.8–200 µM) containing 80 mM glucose. Measurements (*n* = 3) were performed in an open-air environment. Inset: linear correlation between the ΔI_cat_ at either () −0.5 V (slope 511 mA M^−1^ cm^−2^, R^2^ 0.998) or () −0.8 V (slope 326 mA M^−1^ cm^−2^, R^2^ 0.999) and NO_2_^−^ concentration. The supporting electrolyte was 100 mM Tris-HCl (pH 7.6) with 100 mM KCl and 80 mM glucose; scan rate 20 mV s^−1^.

The analyte detection was carried out by covering the SPE-based biosensors with a 50 µL drop of NO_2_^−^ standard solutions (0.8–200 µM) containing glucose, and giving a 5 min incubation time for the scavenger system to remove the dissolved O_2_; CVs were then recorded and the current was measured at the inversion potential −0.8 V (Fig. 6). The increase in NO_2_^−^ concentration resulted in increased cathodic peak currents, according to the EC’ mechanism (see previous section). The ΔI_cat_ varied linearly with NO_2_^−^ concentration in the range of 2–200 µM, with a sensitivity of 326 ± 5 mA M^−1^ cm^−2^ (Fig. 6, inset). The limit of detection (LOD) was estimated to be 4.7 µM, using the standard definition 3*S*_*a*_/*m*, where *S*_*a*_ is the standard deviation of the *y*-intercept and *m* is the slope of the calibration curve.

The sensitivity of the presented biosensor is 35% lower than the one reported in our previous work with c*c*NiR-modified SPE biosensors (550 mA M^−1^ cm^−2^ at −0.8 V working potential)^15^. We attribute the lowered performance to the differences in electrode preparation, namely the absence of carbon conductive ink in the enzyme mixture applied on the WE, and the presence of a protein (GOx/Cat) coat that could be acting as a diffusion barrier to the analyte, thereby lowering the current response. Nevertheless, the analytical features displayed by the biosensor are suitable to monitor NO_2_^−^ in drinking waters according to the guidelines from the World Health Organization (3 ppm or 65 µM)^39^ and the United States Environmental Protection Agency (1 ppm or 22 µM)^40^. In what concerns the European Union directives, the LOD is slightly higher than the maximum permitted value (0.1 ppm or 2 µM)^41^.

Alternatively, the detection of NO_2_^−^ could be performed as well at a less negative potential such as −0.5 V, where the sensitivity was higher (511 ± 11 mA M^−1^ cm^−2^) for a NO_2_^−^ concentration between 0.8 and 150 µM (Fig. 6, inset), with a similar LOD (4.5 µM). Worth mentioning, although the upper limit of the linear range decreased at this working potential, the lower limit was improved.

Another improvement of the new the biosensor configuration to the previous work was the higher reproducibility: RSD 8% at −0.8 V or 4% at −0.5 V (*n* = 10) compared to RSD 20%^15^.

### Storage stability

**Figure 7:**
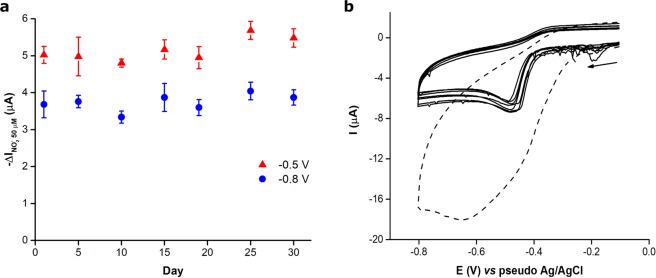
(**a**) Long-term storage stability of a single batch of biosensors recorded over a period of 30 days. Each point represents the average current at () −0.5 V or ()−0.8 V for a 50 µM NO_2_^−^ standard solution (first day *n* = 6; following days *n* = 3). (**b**) Average uncorrected CVs of the SPE-based biosensors’ response to (—) 50 µM NO_2_^−^ standard solution and (╸╸╸) to a blank solution without NO_2_^−^ or glucose. The intense cathodic peak near −0.6 V corresponds to the reduction of dissolved O_2_. The supporting electrolyte was 100 mM Tris-HCl (pH 7.6) with 100 mM KCl; scan rate 20 mV s^−1^.

The long-term storage stability of the developed biosensor was tested over a period of 1 month. To this aim, a batch of single-use biosensors was prepared (those not in use were stored in dry conditions at 4 °C) and the catalytic response to 50 µM NO_2_^−^ was recorded every five days and compared with the one measured on the first day – see Fig. 7a. During this time, the biosensor’s response was considered stable since no consecutive decrease in the ΔI_cat_ was observed, at either −0.5 V or −0.8 V. In Fig. 7b, the average CVs measured in each day are compared with a control bioelectrode (dashed line) tested in electrolyte that did not contain glucose or the analyte. The intense reduction signal of O_2_ observed at −0.6 V in the control is clearly absent from all other CVs, which suggests that the enzymes responsible for removing the dissolved O_2_ from the supporting electrolyte also remain active for 1 month.

### Real sample analysis

Urine (pH 6.75) was selected as the real complex matrix to evaluate the practical application of the proposed SPE-based biosensor in the determination of NO_2_^−^. The biological sample was collected and analysed in the day of the experiments, without any pretreatment. The samples were supplemented with 80 mM glucose and increasing NO_2_^−^ concentrations (0–200 µM). The obtained calibration curve was used to calculate the concentration of the analyte in the real samples. The recovery percentages (Table 1) for the concentrations of 5, 50 and 100 µM were in the range 96–104% when measurements were performed at −0.8 V. These results show that the proposed biosensor could be effectively employed in the determination of NO_2_^−^ in real urine samples where the analyte’s levels have been reported to be up to 400 µM in the case of confirmed bacteriuria^4^.

**Table 1:** Recovery percentages for NO_2_^−^ in urine using the SPE-based biosensor.

NO_2_^−^ added (µM)	NO_2_^−^ found (µM)		Recovery (%)	
	−0.5 V	−0.8 V	−0.5 V	−0.8 V
5	5 ± 2	5 ± 1	101 ± 31	104 ± 23
50	50 ± 2	48 ± 5	99 ± 5	96 ± 9
100	82 ± 7	99 ± 5	82 ± 7	99 ± 5

### Comparison with other miniaturized devices for nitrite determination

The analytical features of the SPE-based biosensor herein presented are summarized in Table 2 and compared with other miniaturized devices developed to monitor NO_2_^−^. Overall, the proposed biosensor performed similarly to the other mentioned analytical tools. Several of these devices are colourimetric paper-based^42–46^ tests, reflecting the current trend of using cellulose materials as substrates for the development of low-cost and sustainable POC assays^47,48^. However, the detection method is mainly based on the Griess reaction^49^, which is rather slow (10–20 min) and prone to interference when used in real samples^9,50–52^, that can only be eliminated by adding a pretreatment step^51,52^. Additionally, special storage conditions (see Table 2) are required to assure the stability of the reactants that are susceptible to photobleaching^43^. The other devices are SPE-based^53–55^ and rely on the oxidation of NO_2_^−^ at high overpotentials, where the oxidation of other common biological molecules could be a source of interference in the analytical assay^52^. Furthermore, some of these devices require complex and high-cost additional components, such as pumps and automatic injectors^53,55,56^, which increase the cost of implementation of the method.

**Table 2:** Comparison of miniaturized analytical devices for the determination of NO_2_^−^.

Device	Detection	LR (µM)	LOD (µM)	RSD (%)	Storage Stability	RT (min)	Ref.
µPAD	Colourimetric (Griess reaction)	Up to 25	5.6	—	12 h at 25 °C (protected from light)	15	^42^
µPAD	Colourimetric (Griess reaction)	87–1800	11.3	—	—	15	^43^
µPAD	Colourimetric (Griess reaction)	10–150	1.0	2.9	1 month at −20 °C (under vacuum)	5	^44^
µPAD	Colourimetric (Griess reaction)	0.7–145	0.43	6.5	—	10	^45^
µPAD	Colourimetric (dihydrotetrazine)	5–500	1.3	3.5–4.7	3 weeks at 4 °C (protected from light)	5	^46^
SPE modified with AgMCs-PPA/PVA	Flow injection amperometry (+0.7 V^a^)	2–800	4.5	<6	—	—	^53^
SPE modified with C*c*R nanostructured layer	Cyclic voltammetry (+0.8 V^a^)	0.1–1600	0.06	3.8	3 weeks at 4 °C	—	^54^
SPE modified with MWCNTs	Batch injection amperometry (+0.7 V^a^)	1–500	0.06	1.1	—	—	^55^
Wireless microfluidic analytical platform	Colourimetric (Griess reaction)	Up to 17	0.49	1.93	—	20	^56^
SPE modified with c*c*NiR/GOx&Cat	CV (−0.5/−0.8 V^a^)	0.8/2–150/200	4.5/4.7	4/8	1 month at 4 °C	5	This work

AgNP-PPA/PVA – silver microcubics-polyacrylic acid/poly vinyl alcohol; c*c*NiR/GOx&Cat – cytochrome *c* nitrite reductase, glucose oxidase and catalase; C*c*R – cytochrome *c* reductase; CV – cyclic voltammetry; LOD – limit of detection; LR – linear range; MWCNTs – multi-walled carbon nanotubes; RSD – relative standard deviation; RT – response time; SPE – screen-printed electrode; µPAD – microfluidic paper-based analytical device.

^a^*versus* Ag/AgCl pseudoreference.

The SPE-biosensor proposed in this work uses a simple and straightforward measurement protocol, without any sample pretreatment, thus being user-friendly and achieving a measurable signal in a short time. Although the device’s substrate is not paper-based, it is made of a thin sheet of plastic material, as opposed to the standard commercially available ceramic SPE, allowing an estimated cost of 0.70 € per biosensor unit.

## Conclusions

In this work, a simple and low-cost procedure for the co-immobilization of an enzymatic O_2_ scavenger system (GOx and Cat) and c*c*NiR (the biosensing element) on a bare carbon SPE was presented. The resulting biosensor was developed for monitoring the enzymatic reduction of NO_2_^−^ in open-air working conditions, without the interference of oxygen. The enzymes GOx and Cat retained their bioactivity upon immobilization, removing dissolved molecular oxygen content from the drop of supporting electrolyte placed on top of the electrode chip, for at least 1 hour. The immobilization of all three enzymes on one electrode allowed for a significant reduction of the work volume required for each measurement, and for a much more practical working procedure, since the need for conventional glass cells and cumbersome oxygen purging methods was eliminated. In terms of analytical performance, the resulting biosensor responded linearly to NO_2_^−^ in the concentration range from 0.8 to 200 µM, with good sensitivity and reproducibility (RSD 4–8%), being well suited for the monitoring of NO_2_^−^ in drinking waters according to international guidelines, and in the analysis of urine in clinical settings. Also, all three immobilized enzymes remained active for at least 1 month.

When c*c*NiR was immobilized on the SPE, interference from CN^−^ (heme-protein inhibitor) on the biosensor’s catalytic response was found to be negligible. Furthermore, the catalase activity was not compromised since no H_2_O_2_ formation was detected. However, with PGE as the immobilization surface, the NO_2_^−^ reductase activity was inhibited in the presence of CN^−^. The carbon ink used for the fabrication of the SPE working and counter electrodes might produce a unique environment for the immobilization of heme proteins, shielding them from CN^−^ inhibition.

This work represents a step forward in the establishment of a truly disposable methodology for low-cost (0,70€ per unit), disposable, on-site NO_2_^−^ biosensing. Furthermore, the immobilized oxygen scavenger system could also be coupled with other reductases, opening a whole new world of possible disposable devices for POC testing where oxygen is a major interferent.
